# Whole-genome sequence of *Kosakonia cowanii* strain W006, isolated from seeds of *Triticum aestivum* L.

**DOI:** 10.1128/mra.01181-23

**Published:** 2024-03-15

**Authors:** Vladimir K. Chebotar, Maria S. Gancheva, Elena P. Chizhevskaya, Oksana V. Keleinikova, Maria E. Baganova, Alexander N. Zaplatkin, Kharon A. Husainov

**Affiliations:** 1 All-Russia Research Institute for Agricultural Microbiology, St. Petersburg, Russia; 2 Department of Genetics and Biotechnology, Faculty of Biology, Saint Petersburg State University, St. Petersburg, Russia; 3 Chechen Research Institute of Agriculture, Chechen Republic, Russia; The University of Arizona, Tucson, Arizona, USA

**Keywords:** genome, sequence, *Kosakonia cowanii*

## Abstract

In this study, we sequence, assemble, and annotate *Kosakonia cowanii* strain W006, isolated from seeds of *Triticum aestivum* L. W006 has a single circular chromosome of 4,788,099 bp and 4,466 genes, with a mean G +C content of 56.1%.

## ANNOUNCEMENT

*Kosakonia cowanii* (previously known as *Enterobacter cowanii*) is a Gram-negative bacterium belonging to the family Enterobacteriaceae. In recent years, *K. cowanii* has been identified as a significant phytopathogen, causing a variety of plant diseases. For example, it has been associated with bacterial wilt in patchouli (1) plants, leading to the death of the affected plants. At the same time, some *K. cowanii* strains exhibit important biocontrol activity. Antagonistic bacterium B-6-1 strain was isolated from the surface of a tomato, identified as *Enterobacter cowanii*, and effectively inhibited the occurrence of *Botrytis cinerea* (2). Here, we report the genome of the *K. cowanii* strain that was isolated from seeds of winter wheat (*Triticum aestivum* L.), cv. Bezostaya 100, as described previously (3). Genomic DNA was extracted from a single colony, grown on LB medium (Thermo Fisher Scientific, MA, USA), using the cetyltrimethylammonium bromide-NaCl method (4).

Long-read whole-genome sequencing was performed in the Core Centrum “Genomic Technologies, Proteomics and Cell Biology” at the All-Russia Research Institute for Agricultural Microbiology using a MinION device with a 9.4.1 flow cell (Oxford Nanopore, UK). SQK-LSK109 Ligation Sequencing Kit, and the EXP-NBD104 and EXP-NBD114 Native Barcoding Expansion kits were used to prepare the library.

The Nanopore reads were basecalled using the Guppy (v. 3.3.0) (5) in the high accuracy mode. A total of 282,817 reads were generated with an *N*_50_ of 8,238 bp. The amount of sequenced DNA was 3 µg in 50 microliters. The largest read is 62,793 bp. The reads were assembled by Flye (v. 2.9) (6), and the resulting assembly was corrected four times using Racon (v. 1.3.2) (7) (with parameters -m 8 -x -6 -g -8 -w 500), followed by a single polish using medaka (v. 1.4.3). The quality of the assemblies was evaluated with QUAST (v. 5.1.0) (8). The assembled genome consists of a single chromosome with a length of 4,788,099 bp and an average G + C content of 56.1%, with genome coverage of 281×. Genome annotation of the assembly was performed by the NCBI Prokaryotic Genome Annotation Pipeline v. 6.6 (9). Based on genome annotation, there were 4,466 genes, including 4,252 coding genes and 116 RNA genes. gcType (10) was used for genome analysis. The average nucleotide identity analysis and 16S rDNA sequence-based analysis revealed that the genome of W006 had high similarity with *Kosakonia cowanii* strain 888-76 (GenBank accession no.: GCA_001975225.1). The biosynthetic gene clusters were identified using AntiSMASH 7.0 (11). The software default parameters were used except where otherwise noted. Six putative gene clusters that function to produce bioactive secondary metabolites were identified, including genes for biosynthesis of antibiotic lankacidin C, Gq protein inhibitor depsipeptide FR900359, O-antigen, siderophore enterobactin, and carotenoid.

## Data Availability

This project has been deposited at GenBank under accession number PRJNA1037303. The genome sequence accession number is CP138453. The raw reads were deposited in the NCBI Sequence Read Archive under accession number SRR26748170.
